# Assessment of Early Therapeutic Response to Nitroxoline in Temozolomide-Resistant Glioblastoma by Amide Proton Transfer Imaging: A Preliminary Comparative Study with Diffusion-weighted Imaging

**DOI:** 10.1038/s41598-019-42088-y

**Published:** 2019-04-03

**Authors:** Nisha Kumari, Nishant Thakur, Hye Rim Cho, Seung Hong Choi

**Affiliations:** 1 Department of Radiology, Seoul National University Hospital, Seoul National University College of Medicine, Seoul, 03080 Republic of Korea; 2 Center for Nanoparticle Research, Institute for Basic Science (IBS), Seoul, 00826 Republic of Korea; 3 School of Chemical and Biological Engineering, Seoul National University, Seoul, 00826 Republic of Korea

## Abstract

Amide proton transfer (APT) imaging is a novel molecular MRI technique to detect endogenous mobile proteins and peptides through chemical exchange saturation transfer. In this preliminary study, the purpose was to evaluate the feasibility of APT imaging in monitoring the early therapeutic response to nitroxoline (NTX) in a temozolomide (TMZ)-resistant glioblastoma multiforme (GBM) mouse model, which was compared with diffusion-weighted imaging (DWI). Here, we prepared TMZ-resistant GBM mouse model (*n* = 12), which were treated with 100 mg/kg/day of NTX (*n* = 4) or TMZ (*n* = 4), or saline (*n* = 4) for 7 days for the evaluation of short-term treatment by using APT imaging and DWI sequentially. The APT signal intensities and apparent diffusion coefficient (ADC) values were calculated and compared before and after treatment. Moreover, immunohistological analysis was also employed for the correlation between APT imaging and histopathology. The association between the APT value and Ki-67 labeling index was evaluated by using simple linear regression analysis. The short-term NTX treatment resulted in significant decrease in APT value as compared to untreated and TMZ group, in which APT signals were increased. However, we did not observe significantly increased mean ADC value following short-term NTX treatment. The Ki-67 labeling index shows a correlation with APT value. APT imaging could show the earlier response to NTX treatment as compared to ADC values in a TMZ-resistant mouse model. We believe that APT imaging can be a useful imaging biomarker for the early therapeutic evaluation in GBM patients.

## Introduction

Glioblastoma multiforme (GBM) is the most malignant primary brain tumor in adults and is uniformly fatal. The current standard of care for glioblastoma includes safe surgical resection, radiotherapy and temozolomide (TMZ) treatment^1,2^. The development of resistance to radiotherapy and TMZ is common^3,4^ and the heterogeneous nature of glioblastoma further complicates these therapies^5^. Unfortunately, average survival is still less than 2 years^1^. Surveillance of the tumor response to drug treatment is essential for treatment supervision or to make any decision^6^. Magnetic resonance imaging (MRI) is a standard neuroimaging technique currently used in the clinical evaluation of the response to therapy based on the detection of tumor size^7^. However, subsidiary imaging methods that can determine the drug response and scrutinize drug-target engagement are critically needed to improve opportune clinical patient management.

One attainable approach is the imaging of metabolism. Altered metabolism has been considered a hallmark of cancer and has been recognized as an important mechanism and biomarker for cancer^8^. There are significant alterations in the metabolic profile between healthy and disease brain tissue that can be detected in patients by using MRI. However, monitoring the response to therapies can be challenging because tumor shrinkage is not always observed^9^. Advanced MR techniques such as diffusion-weighted MRI (DWI), perfusion MRI, and 1 H magnetic resonance spectroscopy (MRS) have also been used to monitor brain tumor response to therapy^10–12^, but it may take several weeks to detect the response to therapy. Therefore, it is imperative to develop novel imaging modality that can easily characterize the progressive tumor and the early therapeutic effect that would help to make decision for ineffective and respondent treatment. Proteins perform many cellular activities, and various lesions, such as those found in tumors, may show changes in the concentration and properties of proteins and peptides^13^. Therefore, information at the protein level may be relevant for earlier detection, better spatial definition, and improved characterization of diseases^14,15^. Amide proton transfer (APT) imaging, one subset of chemical exchange saturation transfer (CEST) imaging, has been introduced as a potentially useful technique that reflects cellular protein and the physical and chemical properties of tissue with image contrast provided by using endogenous mobile proteins or peptides and information on the pH of the tissue^16–18^ that is not available via conventional MRI measures. In addition, APT asymmetry values have been proposed as prognostic indicators of brain glioma, as they reflect the cellular proliferation levels that correlate with Ki-67^19^ and as sensitive biomarkers of treatment response in experimental and clinical studies^20^.

Nitroxoline (NTX) is an FDA-approved antibiotic repurposed for cancer. Lazovic *et al*. revealed that NTX induces apoptosis and slows glioma growth *in vivo*, resulting in a significantly increased apparent diffusion coefficient (ADC) value in a PTEN/KRAS glioma model as determined by DWI following NTX treatment (80 mg/kg/day) for 14 days^21^. We speculated about other imaging modalities with the ability to assess early therapeutic effects of drugs that may help to determine whether the treatment is effective and should be continued or not. The purpose of this preliminary study was to evaluate the feasibility of APT imaging in monitoring the early therapeutic response to NTX in a TMZ-resistant GBM mouse model, which was compared with DWI. We also correlated APT imaging parameters with histopathological findings.

## Results

### Comparison of early evaluation of NTX-mediated therapeutic effects by APT and ADC

**Figure 1:**
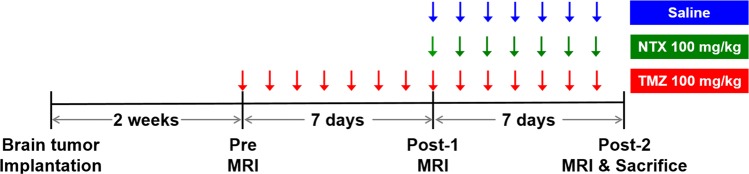
Experimental design for the *in vivo* study, showing the timeline of each group for LN229 cell inoculation, TMZ and NTX therapy, MR imaging, and animal sacrifice for brain harvest as described in the “Materials and Methods” section.

**Figure 2:**
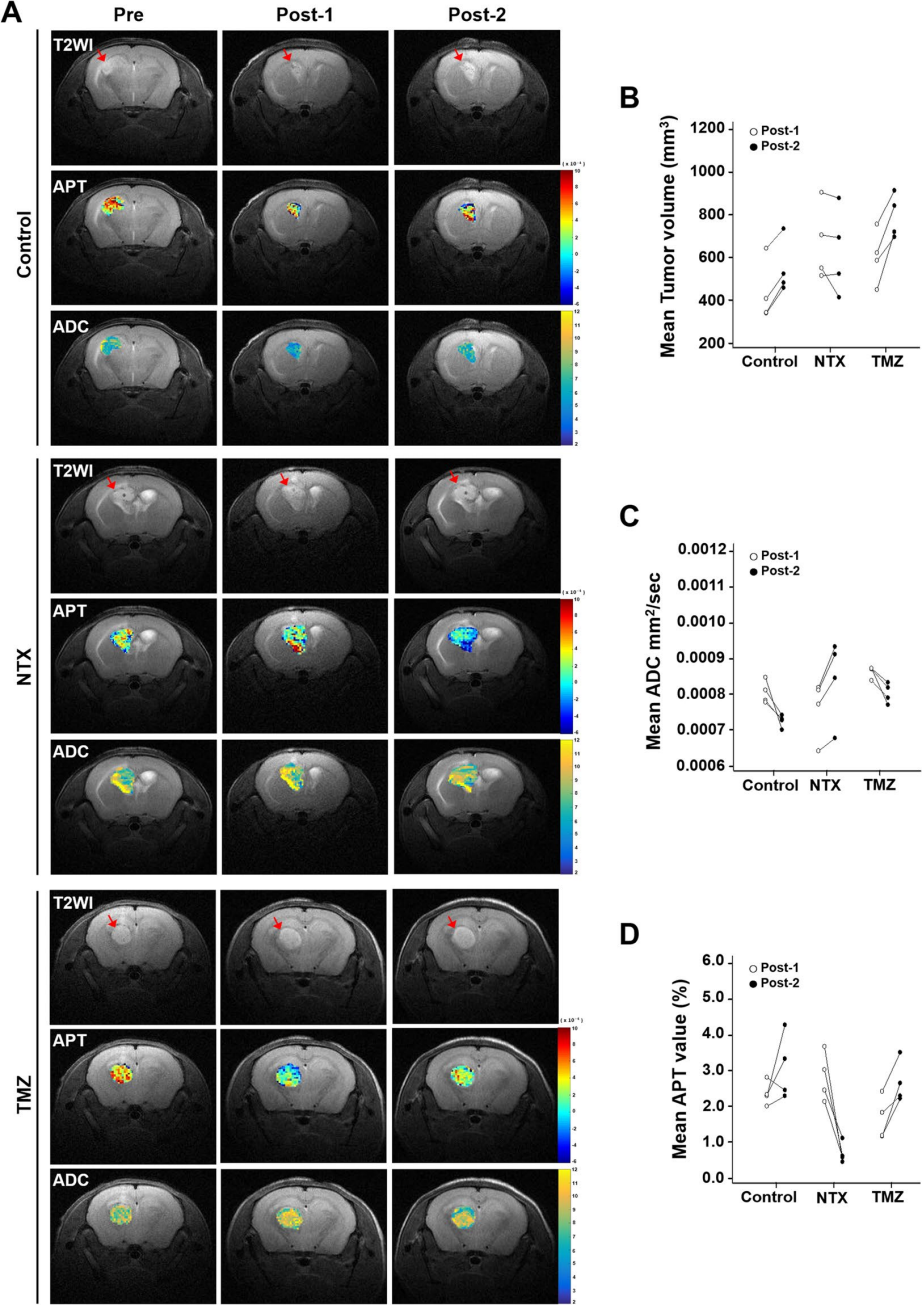
Comparison of the early evaluation of NTX-mediated therapeutic effects by APT and ADC (**A**) Representative T2WIs (1^st^ row), APT maps (2^nd^ row) and ADC (3^rd^ row) for control group, T2WIs (4^th^ row), APT maps (5^th^ row) and ADC maps (6^th^ row) for NTX group, and T2WIs (7^th^ row), APT maps (8^th^ row) and ADC maps (9^th^ row) for TMZ group, imaged on pretreatment MRI, post-1 and post-2 MRI. (**B**) The tumor volume decreased on post-2 MRI in NTX-treated mice (*P* = 0.6857) without statistical significance, and the control (*P* = 0.2000) and TMZ groups showed an increase in tumor volume without statistical significance (*P* = 0.1143). (**C**) The ADC (mm^2^/sec) value increased on the post-2 MRI after NTX treatment without statistical significance (*P* = 0.2000). However, control group (*P* = 0.0286) and TMZ-treated mice (*P* = 0.0286) showed a significant decrease in ADC value. (**D**) The APT value (%) was significantly decreased after NTX treatment (*P* = 0.0286). However, the APT value was not significantly decreased in the control group (*P* = 0.3429) and TMZ-treated mice (*P* = 0.1143). Data are represented as median with an interquartile range of four independent mice in each group.

Figure 1 summarizes the *in vivo* experiments as described in “materials and methods” section. Figure 2A shows representative T2WI, APT, and ADC maps obtained in mice with TMZ-resistant GBM in control, NTX and TMZ-treated groups prior to and after treatment. We observed that the tumor volume decreased without significance in post-2 MRI compared to that in post-1 after short-term NTX therapy [6.26 (IQR, 5.31–8.04) vs 6.08 (IQR, 4.67–7.85), *P* = 0.6857], while tumor volume increased without significance in post-2 MRI compared to that in post-1 in the control [3.73 (IQR, 3.39–5.24) vs 5.01 (IQR, 4.69–6.27), *P* = 0.2000] and TMZ groups [6.02 (IQR, 5.17–6.87) vs 7.79 (IQR, 7.07–8.76), *P* = 0.1143] (Fig. 2B). In terms of DWI, we did not observe a significantly increased mean ADC value (showed in ×10^−3^ mm^2^/sec) in post-2 MRI following short-term NTX treatment [7.91 (IQR, 7.07–8.15) vs 8.79 (IQR, 7.61–9.23), *P* = 0.2000]. In contrast, the mean ADC value was significantly decreased on post-2 MRI in untreated [7.97 (IQR, 7.80–8.29) vs 7.29 (IQR, 7.14–7.35), *P* = 0.0286] as well as TMZ-treated mice [8.71 (IQR, 8.54–8.72) vs 8.03 (IQR, 7.80–8.25), *P* = 0.0286] (Fig. 2C). Interestingly, the significantly decreased APT values were observed in post-2 MRI in NTX-treated mice [2.74 (IQR, 2.29–3.34) vs 0.59 (IQR, 0.50–0.85), *P* = 0.0286]. The APT value was increased in post-2 MRI compared to that in post-1 in untreated mice [2.31 (IQR, 2.15–2.57) vs 2.88 (IQR, 2.37–3.80), *P* = 0.3429] as well as in TMZ mice [1.49 (IQR, 1.16–2.11) vs 2.47 (IQR, 2.26–3.08), *P* = 0.1143] (Fig. 2D).

### Quantification of immunohistochemistry studies

**Figure 3:**
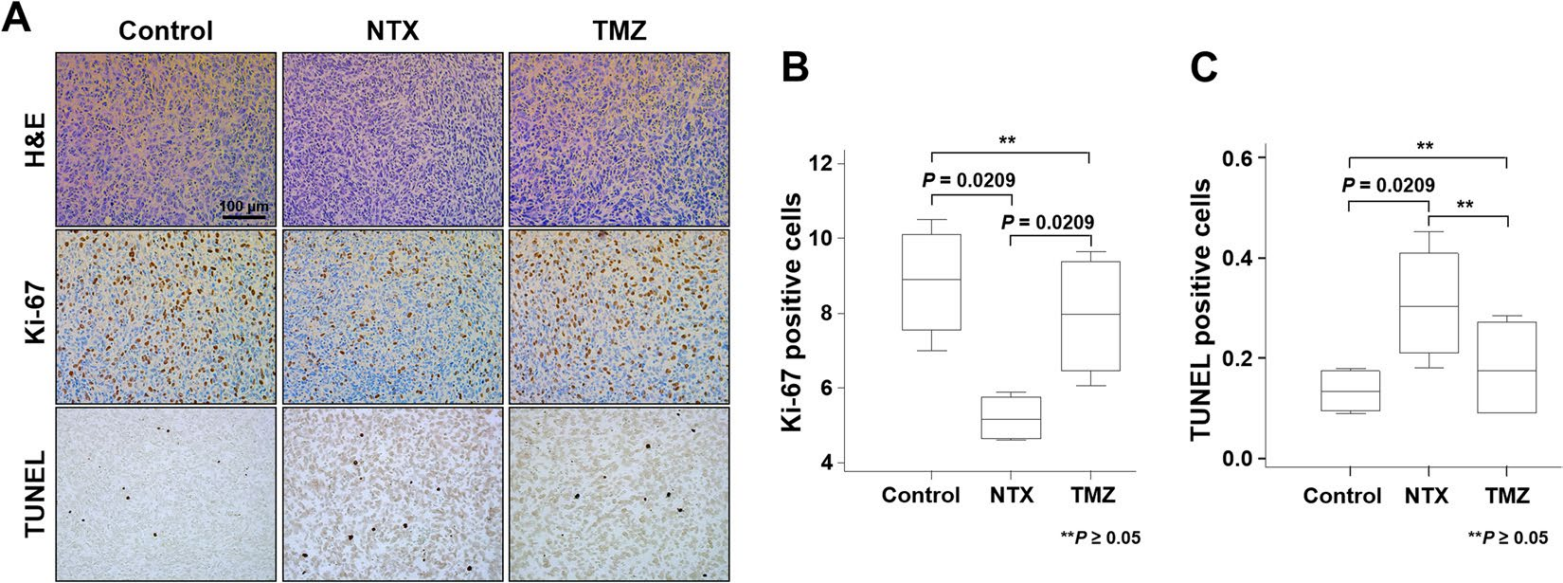
Quantification of immunohistochemistry studies. (**A**) Histological images representing hematoxylin and eosin staining for morphology of tumor (1^st^ row), immunohistochemistry images of Ki-67 for cellular proliferation (2^nd^ row), and TUNEL staining for DNA damage (3^rd^ row) for control, NTX, and TMZ group, respectively. Scale bar is 100 µm in ×200. (**B**) Significantly decreased expression of Ki-67 reflects the decreased cell proliferation in NTX-treated animals (*P* = 0.0209) compared with the control. The Ki-67 expression was significantly increased in TMZ-treated mice (*P* = 0.0209) compared to that in NTX-treated mice. No significant difference was observed between control and TMZ-treated mice. (**C**) Significantly increased TUNEL positive cells reflects the increased DNA damage in NTX-treated animals (*P* = 0.0209) compared with that in the control animals. No significant changes in DNA damage were observed between untreated and TMZ-treated mice (*P* = 0.5637) or NTX and TMZ-treated mice (*P* = 0.2482). Data are represented as median with an interquartile range of four independent experiments.

**Figure 4:**
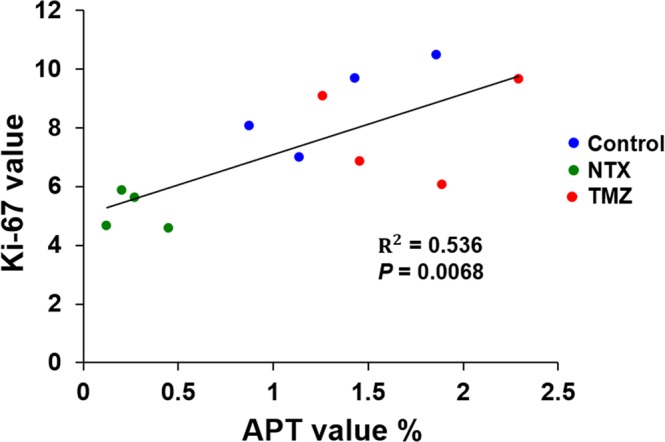
Correlation between the APT and Ki-67 labeling index. Ki-67 showed a positive correlation (R^2^ = 0.536) (*P* = 0.0068) (*n* = 4, per group) with the APT value in all experimental groups.

Based on our *in vivo* results, we performed immunohistology to identify the microscopic changes in tumors for the correlation between APT signals and histological findings. The histological examination of H&E, Ki-67 and TUNEL stained brain sections from NTX-treated, TMZ-treated and untreated TMZ-resistant GBM bearing mice were used to confirm the morphology of tumors, proliferation, and apoptosis (Fig. 3A). The tumor proliferation index was evaluated by the protein expression of Ki-67, which was significantly lower in the NTX-treated group than in untreated mice [control: 8.89 (IQR, 7.55–10.10) vs NTX: 5.14 (IQR, 4.63–5.75), *P* = 0.0209]. The level of cell proliferation was significantly increased following TMZ treatment compared to that following NTX treatment [TMZ: 7.98 (IQR, 6.46–9.38) vs NTX: 5.15 (IQR, 4.63–5.75), *P* = 0.0209)]. There was no significant difference in cell proliferation between TMZ-treated and untreated animals [control: 8.89 (IQR, 7.55–10.10) vs TMZ: 7.98 (IQR, 6.46–9.38), *P* = 0.2482)] (Fig. 3B). Furthermore, the number of apoptotic cells were investigated by TUNEL assay staining in which a significantly increased number of apoptotic cells were observed following NTX treatment [control: 0.13 (IQR, 0.09–0.17) vs NTX: 0.30 (IQR, 0.21–0.41), *P* = 0.0209] compared to that following the control. However, there was no significant difference of apoptosis between the untreated and TMZ-treated animals [control: 0.13 (IQR, 0.09–0.17) vs TMZ: 0.17 (IQR, 0.09–0.27), *P* = 0.5637] or between the NTX- and TMZ-treated mice [NTX: 0.30 (IQR, 0.21–0.41) vs TMZ: 0.17 (IQR, 0.09–0.27), *P* = 0.2482] (Fig. 3C). Furthermore, we observed that the Ki-67 labeling index between all experimental groups showed a correlation with APT value (R^2^ = 0.536, *P* = 0.0068) (Fig. 4).

## Discussion

In this preliminary study, we employed two techniques, APT imaging and DWI, to assess the early therapeutic effects of NTX in a TMZ-resistant GBM mouse model. APT and ADC values were analyzed. APT imaging (a subtype of CEST imaging, sensitive to amide protons resonating at 3.5 ppm from water) was designed to detect mobile (cytosolic) proteins and peptides^22^. Malignant gliomas are highly cellular and have a higher cellular content of proteins and peptides than healthy tissue, as revealed by MRI-guided proteomics and *in vivo* MR spectroscopy^23,24^ and it has been assumed that amide proteins of endogenous mobile proteins and peptides in the cytoplasm are the major source of the APT signals^22^. In our study, we determined that APT imaging can detect metabolites that are elevated or down-modulated after NTX treatment. Short-term NTX treatment resulted in a substantial reduction in APT signals, although the tumor volume and ADC value did not detectably change by T2WI and DWI. The subsequent increase in APT value in the untreated group and TMZ-treated mice indicates the further progression of the tumor.

Generally, successful treatment may cause necrosis or apoptotic processes, leading to a reduction in cell density, which would increase the ADC values. Several studies have reported that the ADC is useful to estimate the aggressiveness of many cancers and correlates with progression-free survival^25,26^. However, in other reports, ADC was limited to malignancy evaluation, because a minimal change of ADC value cannot reflect the metabolic changes or changes in cellularity after early exposure to chemotherapeutic agents^27–29^. In our study, ADC value did not detectably change after NTX treatment by DWI. However, the ADC value was significantly decreased in the control and TMZ groups, which suggests increased cellular proliferation in the tumor and possibility of ADC value application for non-responder. The undetectable change in ADC value was not sufficient to evaluate the early response to NTX, while the APT value was dramatically decreased after NTX therapy. The undetectable change in mean tumor volume following short-term NTX treatment indicates that compared to no treatment and TMZ treatment, NTX slows down tumor growth. This result was similar to that of a previous study^21^.

Although TMZ, a second-generation alkylating agent, is considered one of the best choices for standard adjuvant treatment for GBM, it is a DNA-damaging agent and induces apoptosis. One might assume that the 1-week exposure may be sufficient to induce the APT changes in the NTX-treated group, which could be associated with apoptosis within the 7 days. To further scrutinize this possibility, we investigated the TMZ group in which we observed the increased tumor volume and APT values after 2 weeks of TMZ treatment. Moreover, the change in ADC value was subsequently investigated in which we observed significantly decreased ADC values after TMZ treatment. This finding clearly reflects the further progression and the development of resistance to TMZ treatment.

Histological examination of the tissue showed that the 2-week TMZ treatment induced a marked increase in Ki-67 expression, which results from increased mitosis. According to the relevant literature, the APT signals of brain tumors are positively correlated with cellular density and proliferation, arising from intracellular mobile proteins and peptide^19,30^. Similar to previous reports, we showed the positive correlation between APT signals and cellular proliferation as evaluated by Ki-67 staining. This observation suggests that APT signals reflect the production of endogenous mobile proteins and peptides, which is associated with tumor cell proliferation. Therefore, from this observation, we concluded that the decrease in APT values observed in NTX-treated mice reflects the proliferation arrest of tumor cells responding to chemotherapy well before the tumor volume and cellular density begins to decrease. As molecular changes occur early in the time course of chemotherapy, preceded by morphologic changes, which cannot be detected by conventional MRI; thus, APT signals may serve as an early biomarker of the degree of tumor progression or treatment response. Increased APT signals following TMZ treatment indicates the aggressiveness of recurrent GBM even after chemotherapy with TMZ. In relation to TMZ-resistance, the enhanced tumor area increased drastically after 1 week of TMZ treatment compared to that at baseline, indicating that TMZ-resistance was acquired as showed by the post-1 MRI. The results also demonstrate that APT signal does not decrease when the treatment is ineffective in case of TMZ-treated animals.

The limitations of this study should be mentioned. First, we included only four mice in all experimental groups. Our institutional policy is to minimize enrolled animal numbers; thus, we decided to conclude our study after observing statistical significance. Second as APT imaging is a rapidly developing field, the imaging protocols have not yet been fully standardized, and it is warranted to confirm the reproducibility of APT imaging of brain tumor. Thus, the future studies regarding these issues should be performed. Third, we used six b-values for DWI in this study, which need longer time than the common clinical DWI based on two b-values, because we wanted to analyze other DWI-related parameters such as perfusion values, which were not shown in this study. We believe that DWI using two b-values can be enough for future study to minimize scan time without any compromised data quality. Fourth, we used T2WI to define the tumor areas, which has limitation to differentiate the peritumoral edema from tumor invasion. However, we think that the high intensity change on T2WI is mainly from the tumor growth, which was correlated with the histopathology.

In summary, we concluded that compared to ADC values, APT imaging could show an earlier response to NTX treatment in a TMZ-resistant mouse model. APT imaging is a safe, completely noninvasive technology and we believe that APT imaging can be a useful imaging biomarker for the early therapeutic evaluation in GBM patients.

## Material and Methods

### Study design

The animal experiments were approved by the Institutional Animal Care and Use Committee of Seoul National University Hospital and used minimum number of mice to conclude with statistical analysis. All research was performed in accordance with relevant guidelines/regulations in our institute (16–0130-C1A0). The *in vivo* experiments were performed according to Fig. 1. To prepare for the orthotopic GBM mouse model, 6-week-old male BALB/c nude mice (*n* = 12, 4 in each group) were anesthetized by means of intraperitoneal injection of a mixture of Zoletil (zolazepam) and Rompun (xylazine) and were placed in a stereotaxic device. The mice were inoculated with LN229 (ATCC, CRL-2611) human glioma cells (3 × 10^6^ cells). The cells were injected in the caudate/putamen region of the brain by using a Hamilton syringe fitted with a 28-gauge needle, which was positioned with a syringe attachment fitted to the stereotaxic device. The required tumors were confirmed by pretreatment T2WI 2-weeks after tumor implantation. TMZ-resistant models were developed by subjecting mice with GBM to successively high doses of TMZ (100 mg/kg/day) until tumor growth showed no inhibition by TMZ for 7 days, as described in previous studies^3,31,32^. The models were further confirmed by T2WI, referred to as the post-1 MRI. The animals were intraperitoneally treated with 100 mg/kg/day of NTX or saline for 7 days in the NTX and control groups, respectively. In the TMZ group, the animals were treated with 100 mg/kg/day of TMZ for 7 days after post-1 MRI. The post-2 MRI was conducted after drug treatment to evaluate the therapeutic effects on tumor growth. APT imaging and DWI were also acquired sequentially with T2WI in pretreatment, post-1 and post-2 MRIs.

### MR imaging protocol

For the *in vivo* animal MRI, animals were anesthetized with 1.5–2% isoflurane/oxygen (v/v), and then scanned using a 9.4 T MR scanner (Agilent Technologies, Santa Clara, CA, USA). Throughout each imaging session, the animals were wrapped in warm water blankets, and their oxygen saturation and heart rates were monitored. In anatomic T2WI in the coronal plane, fast spin-echo multiple slices were employed with the following parameters: [TR = 3000 ms, effective TE = 31.18 ms, ETL = 4, averages = 2, data matrix size = 256 × 256, and field of view (FOV) = 25.0 × 25.0 mm^2^]. APT imaging with coronal plane was performed by using a prototype 2-dimensional saturation pulse target frequency 3.5 ppm, saturation pulse duration 5 s, slice thickness 1.00 mm, saturation pulse power 20 Hz followed by four-shot, spin-echo, echo-planar imaging acquisition [TR = 5500 ms, TE = 9.34 ms, ETL = 4, averages = 1, data matrix = 128 × 128, field of view (FOV) = 25.0 × 25.0 mm^2^]. The echo-planar DWI with coronal plane was obtained as followings: [TR = 4000 ms, TE = 60.04 ms with shots 2, repetitions = 1, average = 2, data matrix = 128 × 128, field of view (FOV) = 24.0 × 24.0 mm^2^, b-value = 0, 100, 200, 400, 700 and 1000 s/mm^2^, and slice thickness = 1 mm]. The APT and ADC maps were generated, and image analysis was performed by using our in-house software developed with a commercial analysis package (Matlab version R2007b, MathWorks Inc., Natick, MA, USA).

### Image analysis

To quantify the tumor volume, the areas on the ADC and APT maps corresponding to the T2W images were first manually segmented by two investigators (N.K. and N.T.). The segmentations of ADC and APT maps were performed for all the slices showing T2 hyperintensity. The identification of corresponding area with T2 hyperintensity on ADC and APT maps was performed by visual inspection, and then polygonal ROIs were placed on coregistered ADC and APT maps for corresponding area with T2 hyperintensity. Drawing ROIs were performed with MATLAB (Matlab version R2007b, MathWorks Inc., Natick, MA, USA) and systematically placed. Then, we calculated tumor volume, and mean APT and ADC values from each tumor. The change in mean ADC values was included in the analysis.

### Histology analysis

At the end of the post-2 MRI studies, all animals were sacrificed and perfused with normal saline, and brains were harvested for histological analysis. All coronal sections were stained with hematoxylin and eosin (H&E) for tumor morphology. For immunohistochemical analysis, the primary antibody and their dilutions were as follows: mouse monoclonal antibody to Ki-67 (1:200, no. UM 8033, UltraMab) was used for 1 hour at room temperature. Then, the sections were rinsed with washing buffer and incubated with horseradish peroxidase-conjugated secondary antibodies (Santa Cruz Biotechnology) for 30 minutes at room temperature. Staining for the detection of bound antibodies was evaluated by DAB. Ki-67-positive cells and TUNEL-positive cells were calculated by ImageJ software. Additionally, brain tumor sections were subjected to TUNEL assay to measure apoptotic tumor cells.

### Statistical Analysis

All statistical analyses were performed using a commercial software program (MedCalc version 13.1.0.0, MedCalc Software). A *P* value of <0.05 was considered statistically significant. Kolmogorov-Smirnov’s test was used to determine whether the noncategorical variables were normally distributed. Nonparametric data are presented as the median and interquartile range (IQR, range from the 25th to the 75th percentile), and parametric data are shown as the mean ± standard deviation. According to the results of the Kolmogorov-Smirnov’s test, paired or unpaired Student’s t-test, Wilcoxon test or Mann-Whitney U-test was performed where appropriate to compare the values between the two groups. The comparison between the three experimental groups was calculated by the Kruskal-Wallis test. The association between the APT value and Ki-67 labeling index was evaluated by using simple linear regression analysis.
